# Cross-cultural assessment of prolonged grief symptomatology in Switzerland, Rwanda, and Viet Nam: protocol for an experience sampling study

**DOI:** 10.1186/s40359-025-03682-0

**Published:** 2025-11-28

**Authors:** Huy Hoang Le, Pelin Sila Gelmez, Celestin Mutuyimana, Eva-Maria Stelzer, Hung Nguyen, Clare Killikelly

**Affiliations:** 1 Department of Psychology, Division of Clinical Intervention and Global Mental Health, University of Zurich, Zurich, Switzerland; 2 School of Science Engineering and Technology, RMIT University Viet Nam, Ho Chi Minh city, Viet Nam

**Keywords:** Grief, Bereavement, Experience sampling method, Ecological momentary assessment, Daily assessment, Digital screening, Digital mental health, Cross-cultural comparison

## Abstract

**Background:**

Despite grief being a universal experience, current understanding of the trajectory and predictive factors of the new condition, Prolonged Grief Disorder (PGD), mostly arises from studies conducted in Europe and North America. Research suggests cultural variations in how grief develops across time. This study aims to longitudinally compare PGD growth patterns across Swiss, Vietnamese, and Rwandan bereaved individuals.

**Methods:**

Due to grief’s wave-like nature, experience sampling methods (ESM) enables a fine-grained temporal analysis of grief manifestation. A total of 100 bereaved individuals from each country who lost a loved one in the last 3 years will complete a 2-week ESM assessment app (mPath) every 3 months for 6 months (Viet Nam and Rwanda) or 18 months (Switzerland). The app sends daily reminders to complete a questionnaire about PGD symptoms and daily life context. Participants will also complete a baseline mental health assessment before each ESM period. The primary outcomes are symptom variability and chronicity, while secondary outcomes include culturally specific predictive factors of prolonged grief symptoms (e.g., mourning rituals). Acceptability and feasibility of daily assessment will also be assessed. The method and specific ESM items have been developed and tested through cognitive interviews with bereaved individuals from each cultural group to ensure cultural acceptability.

**Discussion:**

The current study is the first to longitudinally explore potential cultural variations in grief trajectories across Swiss, Vietnamese, and Rwandan bereaved individuals. This study has significant potential to contribute to more nuanced, culturally informed, and technologically integrated approaches to bereavement care.

## Background

Although losing a loved one is a universal experience, a minority of bereaved individuals are confroted with an increased risk of trauma and stress-related mental health disorders [1]. Prolonged Grief Disorder (PGD) is a recently recognized mental health condition, officially included in the Diagnostic and Statistical Manual of Mental Disorders, Fifth Edition, Text Revision (DSM-5-TR) [2] and International Classification of Diseases 11th Revision [3]. Unlike typical grief, PGD is an atypical and persistent grief response characterized by intense and enduring yearning or preoccupation with the deceased, emotional pain, identity disturbance, loss of meaning and purpose, and various cognitive, physical, or behavioral disruptions [4]. These symptoms lead to significant functional impairment that exceeds expected social, cultural, or religious norms [4, 5].

Current estimates suggest that for each deceased person, approximately nine family members will face emotional and psychological issues; of these, about 60% recover naturally, while 40% are at risk of developing mental health issues [6]. Within this group, roughly 10% experience persistent, severe, and debilitating grief ([7, 8]. A recent study estimated the global prevalence of PGD at 13% [9], with middle-income East Asian countries reporting lower rates (9.2%) compared to Western countries (10.2%) [10]. In Asia, most of the studies reported on the prevalence of PGD were carried out in China, reporting an average of 8.9% [11]. While there has not been an estimate for PGD in Viet Nam, it is estimated that over 155,000 bereaved individuals are likely to require grief support after the COVID-19 pandemic [12]In Africa, a few existing studies reported varying prevalence rates. For example, a recent study comparing three African countries—Kenya, South Africa, and Namibia found relatively high prevalence rates of 9.63% in Kenya, 6.85% in South Africa, and 5.34% in Namibia [13]. The varying prevalence rates of diagnosis in different countries can be accounted for by cross-cultural differences in the expression, perception, and management of grief [14–17]. For example, a study showed that feelings of loss and difficulty moving on were central symptoms in samples from Ghana and Nigeria, while core symptoms such as preoccupation and longing were less prevalent [18]. Additionally, bereaved Cambodian refugees hallucinate and dream of the deceased [19]. Culturally specific expressions, also known as idioms of distress, have been found to have clinical utility in identifying psychopathologies in non-Western countries [20, 21]. Accurate diagnosis must then consider cross-cultural differences in the expression, perception, and management of grief [14].

Such a discrepancy is echoed by the global mental health vulnerability paradox, where Global North countries with greater mental health care resources reported higher rates of mental health issues [9]. Within the context of losing a loved one, country vulnerability is associated with collective beliefs influencing the experience of social support and the risk of PGD [9]. This points to recent evidence suggesting traditional mourning rituals buffer against post-loss psychological consequences by promoting and fostering communal support in African and East Asian countries [22, 23]. Alternatively, restricted mourning due to COVID-19 social restrictions was found to be associated with heightened risks for spiritual concerns (e.g., ghost visits, loss of faith, etc.) and distressing somatic symptoms [24–28]. Although there has been a shift toward multicultural comparative grief research, methodological heterogeneity and cultural variations have posed significant barriers to reliable global findings [17, 29].

Researchers must assist clinicians in differentiating PGD from not only normal grief but related mental health problems [30, 31]. For instance, approximately 13% of bereaved individuals develop PGD, 12–16% exhibit posttraumatic stress symptoms, and 19% meet depression diagnostic criteria [9, 32, 33]. A recent meta-analysis further confirmed that among those with clinical PGD symptoms, 40% have comorbid PTSD and 63% experience depression [34]. However, the evidence regarding the trajectory of bereavement remains inconsistent. Some studies indicate that early PGD symptoms predict later psychopathology, including post-traumatic stress disorder (PTSD), adjustment disorder, mood disorders, and anxiety disorders [35–40]. Yet other findings suggest the reverse, with PTSD symptoms preceding or predicting PGD, pointing to a bidirectional relationship between these conditions [4, 41]. Although early interventions can benefit depression and PTSD, there are negative impacts when natural grieving processes are disrupted prematurely [42, 43]. A better understanding of how PGD unfolds is needed for an accurate and timely diagnosis of PGD to provide appropriate interventions. This underscores a need for improved methodological precision in studying grief trajectories, highlighting the importance of approaches that can capture the dynamics of PGD and related symptoms.

Building upon previous claims of ‘waves and ‘pangs’ in everyday life context [44], the app–based experience sampling method (ESM) has proven to be a feasible platform to monitor early prolonged grief in everyday life [45–47]. ESM involves real-time tracking experiences, usually through self-reports, in the real world [48]. This approach allows for early detection of mental health deterioration through insights on symptom triggers and progression [49–51]. Additionally, not only do existing ESM grief studies not report any negative reactivity effort, but bereaved participants also welcomed the opportunity to discuss their grief experiences [45, 47].

The field of grief research is currently at a critical turning point, where clinicians and researchers are urgently called upon to provide treatment and support for those bereaved ever since the pandemic, while also adapting to the introduction of a newly defined grief-related disorder. Currently, large-scale multi-site research on the psychological consequences of bereavement remains limited. Building on previous concerns about inaccuracies in prevalence rates, diagnostic criteria, symptom variations, and cultural influences, cross-cultural research provides a timely holistic understanding of PGD manifestations.

## Methods/design

### Aims and research questions

This study aims to conduct a cross-cultural assessment of PGD symptomatology in three countries: Switzerland, Rwanda, and Viet Nam. We will answer the following research questions:

1. What are the dynamic patterns and determinants of grief trajectories in daily life?
  1.1. What are personal, loss-related, and situational factors associated with elevated grief symptoms? How do they predict grief trajectories over time?
  1.2. How stable or variable are core and accessory symptoms of grief across time and individuals?
2. How do culturally specific support systems, mourning behaviors, coping strategies, rituals, and traditions shape the experience and course of grief across Switzerland, Rwanda, and Viet Nam?
  2.1. Does participation in local mourning rituals decrease the likelihood of higher grief scores?

### Study design

This study employs a multi-site longitudinal design using ESM to examine daily grief processes and cultural influences on grief trajectories in bereaved adults across Switzerland, Rwanda, and Viet Nam. The study combines repeated intensive ESM assessments with baseline and follow-up surveys to capture both moment-to-moment fluctuations and long-term patterns of grief symptoms and adaptation.

### Participants

100 adults (18 years or older) from each research site who experienced the death of a close person (e.g., family members, spouse, close friends, etc.), have access to the internet and a smartphone, and are currently not using any formal mental health services, will be recruited in each country. Participants are grouped into two categories: early bereaved (3–6 months since the loss) and later bereaved (6 months – 3 years since the loss).

### Recruitment

Purposive sampling will be used to identify bereaved individuals. Participants will be recruited through the local community networks by asking key contacts from organizations and networks to distribute and post our recruitment ads through online platforms (e.g., WhatsApp groups, Facebook groups, etc.), through community networks (online community forums and face-to-face networking at local organizations). Contact letters will be sent to student organizations. Flyers and posters will also be posted around universities and at local community centers, libraries, hospitals, hospices, and other public places with permission.

###  Materials

The survey includes demographics, loss-related questions (e.g., date of death, relationship to the deceased, cause of death, closeness to the deceased, and any mourning rituals performed), the Patient Health Questionnaire (PHQ-9) [52]; the Generalized Anxiety Disorder-7 (GAD-7) [53]; the 8-item Somatic Symptom Scale (SSS-8) [54]; World Health Organization Wellbeing Index (WHO-5) [55]; the International Trauma Questionnaire (ITQ) [56]; the International Prolonged Grief Disorder Scale (IPGDS) [57]; the Oxford Grief Social Disconnection Scale (OG-SD) [58]; Depressive and Anxious Avoidance in Prolonged Grief Questionnaire (DAAPGQ) [59]; MyGrief scale (O’Connor et al., unpublished manuscript); Reactions to Research Participation Questionnaire adapted for grief-ESM research [46], and one item for assessment of need of psychological support developed for this study (“Has your grief in the past two weeks led you to consider seeking professional help?”).

The daily ESM questionnaire consists of 3 items from the IPGDS [57] and 9 MyGrief items (O’Connor et al., unpublished manuscript), 3 daily life context questions (e.g., “What were you just doing?“; “Where are you right now?“; “Who are you with right now?“), and 3 enjoyment questions related to activity, place, and person (e.g., “How much do you enjoy this activity?”; “How much do you enjoy being here?”; “How much do you enjoy being around this person?”).

In this study, we use the digital app m-Path (www.mPath.io/dashboard) as the platform for our smartphone-based ESM. m-Path offers an easy-to-use and highly customizable framework. To gather baseline data, we conduct an online assessment using LimeSurvey (http://limesurvey.org).

###  Procedure

The research team will implement a standard translation process for psychological tests to ensure the quality of the questionnaire [60]. Two independent translators, one fluent in the native language of their respective country (e.g., Vietnamese, German, French, etc.) without a psychology background, and the other fluent with psychological expertise, will perform forward and back-translations, respectively. A panel of experts with psychological expertise will review and compare the translations for accuracy and appropriateness.

Cognitive validity interviews will be conducted for the novel ESM questionnaire at the three study sites to ensure item validity and identify potential misalignments between participants’ and researchers’ interpretations. These interviews will also evaluate the clarity and language adaptation of the ESM items, determining their understandability and cultural appropriateness. Researchers will conduct online focus groups with bereaved adults from each country using convenience sampling. They will answer questions formulated based on Peterson and colleagues’ [61] cognitive operations: “1) Do you understand intuitively the question? If no, why? 2) Is there any difficult word? If yes, can you propose another word in your language? 3) Is there any word we can change?; 4) Do you find similar symptoms in people who suffer from loss in your culture?; 5) If you compare all items, which one was too easy, which one was too difficult, and why?; 6) Is there any sensitive item which could be reformulated?” Based on the comments and suggested changes, the research team will consider adjusting the questions to make them more understandable and culturally appropriate.

**Fig. 1:**
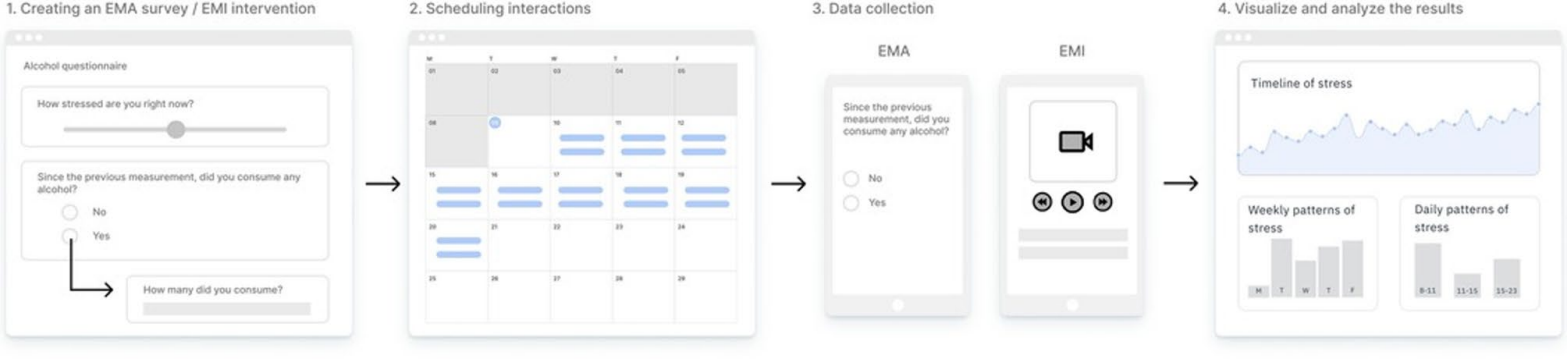
The m-Path workflow. First, researchers create and fine-tune the ESM survey in the interaction editor (Panel 1). Next, these ESM interactions are scheduled in the calendar view (Panel 2). Third, participants receive notifications on their personal smartphones to interact with the ESM content (Panel 3). Finally, researchers can analyze incoming results of a single participant in real-time via modifiable graphs and charts in the online dashboard (Panel 4). Reproduced from Mestdagh, Verdonck, Piot, Niemeijer, Kilani, Tuerlinckx, Kuppens, and Dejonckheere, 2023, *Frontiers in Digital Health*, 5:1182175 [62] under the terms of the Creative Commons Attribution License (CC BY)

To schedule daily assessments, researchers log into m-Path’s online dashboard (www.m-Path.io/dashboard) using their existing account. This allows them to create an ESM template (Protocol) using validated questions for future participants. For more information, refer to Fig. 1, which illustrates our m-Path workflow [62].

### Data collection

#### Step 1: usage instructions

Following a screening phone call to ensure study eligibility, participants will be shown how to download and use the mPath application. They will complete a brief trial run of the app (5 min). We will also send a follow-up email with detailed instructions for reference. Participants will then be invited to participate in the baseline assessment and will sign an online consent form via the LimeSurvey assessment tool.

#### Step 2: baseline assessment(PreESM)

Before each ESM survey, participants will be required to complete an online assessment on Lime Survey. The assessment will take about 1 h. Vietnamese and Rwandan participants will complete this assessment two more times, after 3 months and 6 months. German-speaking participants in Switzerland will complete assessments with the full battery of questionnaires every 6 months (e.g., 6 months, 12 months, 18 months) and a reduced battery of questionnaires in between (e.g. 3 months, 9 months, 15 months) for 18 months. Suicide risk was assessed during the baseline measurement.

#### Step 3: ESM data collection

Participants will fill out an integrated questionnaire on the m-Path about PGD symptoms and their daily life context 5 times daily (1 in the morning, 1 at noon, 1 in the afternoon, and 2 in the evening) for over two weeks. Participants will complete the mobile app ESM sampling phase of assessment 3 times in total, each lasting 2 weeks, with intervals of 3 months, over 6 months in Rwanda and Viet Nam. In Switzerland, this process will be conducted for 18 months.

### Safety considerations

At the initial setup meeting, participants receive a list of local mental health resources and contact information for two designated study team members available by phone for emergencies. Participants may contact the study team or local services at any time. Suicide risk is screened during the pre-ESM baseline assessment. Participants indicating elevated risk are promptly contacted and, with consent, invited to a follow-up interview with a licensed clinician. Based on clinical judgment, participants may be excluded from further participation and referred to local services if needed. During the ESM period, daily grief scores are monitored, and participants showing significant elevations in PGD items are contacted and referred to appropriate resources with their consent and may be excluded from further participation.

### Data analysis plan

#### Research question 1.1: predictors of temporal fluctuations in grief symptoms

To identify personal, loss-related, and situational predictors of temporal fluctuations in grief, multilevel modeling (MLM) will be used to account for the nested structure of the experience sampling method (ESM) data (repeated measures within individuals). At the within-person level, person-centered ESM scores will capture moment-to-moment variability in grief symptoms. At the between-person level, time-invariant predictors such as age, gender, education, kinship to the deceased, cause and timing of death, and major life events will be entered to estimate their influence on overall symptom levels and variability.

#### Research question 1.2: stability and variability of core and accessory symptoms of grief

To examine how core and accessory symptoms fluctuate over time and across individuals, latent growth curve modeling (LGCM) will be performed using baseline and ESM data. This approach will estimate individual trajectories and test whether symptom changes follow linear or nonlinear trends. Additionally, variance components from the multilevel models will be used to quantify the proportion of within- versus between-person variability in grief symptoms.

#### Research question 2: cultural influences on the experience and course of grief

To assess how culturally specific support systems, rituals, and traditions shape grief trajectories, comparative and multigroup analyses will be conducted across Switzerland, Rwanda, and Viet Nam. First, descriptive and correlation analyses will characterize post-loss support systems and mourning practices within each cultural context. Subsequently, multiple Latent Growth Curve Modeling (MG-LGCM) will test whether the strength and direction of associations among grief symptoms, cultural practices, and adjustment outcomes differ across countries.

## Discussion

### Significance of potential results

Despite being a relatively new mental health condition, PGD has been extensively studied across various cultures to understand its progression and potential comorbidities [24, 36, 63, 64]. Nonetheless, existing discrepancies in diagnostic approaches across multiple cultural settings challenge the understanding of how and why PGD symptoms may vary within and across cultural groups, contributing to the overestimation of PGD worldwide [17, 65]. The study will not only be the first to explore grief trajectories in understudied Global South samples (e.g., Rwanda and Viet Nam) but also to be the first to longitudinally reveal cross-cultural differences in early PGD progression across an international sample.

Although previous evidence proposed yearning and preoccupation as universal hallmarks of PGD [66], recent cross-cultural comparisons found the largest average differences for yearning, sorrow, and guilt [63]. Similarly, although recent ESM grief studies reported changes in yearning and sorrow intensity as the two most robust indicators of daily life experiences [67, 68], daily grief reactions might exhibit similar patterns. That is, it is expected that not all symptoms will fluctuate in the same manner across the three countries; such centrality of yearning and sorrow might only reflect the daily experience of a European sample. Our results are then expected to explore the extent to which daily grief experience vary in response to differences in sociocultural contexts between Switzerland, Rwanda, and Viet Nam.

Our cross-cultural comparison of post-loss trajectory memberships will further elaborate on previous findings of a predominantly resilient outcome [69]. However, Swiss, Vietnamese, and Rwandan bereaved individuals’ memberships of PGD severity (low, mild, high) might differ depending on the nature of their cultural mourning practices. With reference to the framework of cultural scripts of trauma, bereaved individuals might express their strong emotions in alignment with the availability of social support systems and cultural norms [15, 23, 70]. Thus, culturally-scripted mourning rituals constitute a core component of culturally normative grief responses and may play a critical role in shaping the psychological sequelae of bereavement. In consideration of cultural differences in mourning and bereavement, our study includes ritualistic (*“perform memorial rituals e.g.*,* burn incense*,* pray*,* worship*, etc.”) and proximity-seeking behaviors (“*relive memories of the deceased e.g.*,* look at old photos and mementos of my partner”*) in addition to daily life activities. Such rituals and activities might promote post-loss resilience among individuals of interdependent cultures (e.g., ethnic Asians and Africans) since they are usually performed collectively. Hence, constructing an immediate supportive social circle [22, 23] consequently cultivates momentary positive affect to prevent mental health deterioration.

Previous studies have found that under the same diagnostic criteria, Greek bereaved individuals who practice prolonged and communal death rituals months and years after the death reported higher PGD scores compared to Iran, Turkey, and the U.S [63, 71]. Following the same pattern, Swiss bereaved individuals reported lower rates of PGD symptoms compared to the Chinese bereaved sample [40], who practice death rituals for an extended period post-loss [72]. However, these findings are inconsistent with previous research on interdependent cultures (e.g., ethnic Asians and Africans) showing more favorable outcomes for traumatic stress compared to independent cultures (e.g., ethnic Europeans and Australians) [73]. A recent study of the Luhya People of Kenya by also reported that religious rituals post-loss helped them process their grief [22]. Cultural differences in the duration and expression of mourning may explain such variations in grief scores. Therefore, as we control for instrument heterogeneity in this cross-cultural study, potential results could reveal how cultural differences in loss-related behaviors and attitudes may explain differences in reported PGD prevalence and long-term trajectory.

Our daily-life context ESM items will also provide a window into how grief reactions fluctuate in response to the daily life context. Eisma and colleagues [74] found a significant presence of daily negative thoughts and negative affect among individuals with higher prolonged grief symptoms. The findings of previous ESM studies [75, 76] on depression extend the findings of Eisma and colleagues [74], as more sedentary behaviors and less engagement with close social contacts showed elevated levels of end-of-day negative affect. Changes in physical, sedentary, and social behaviors throughout the day could predict end-of-day depressive symptoms in addition to previous-day affect symptoms. Several studies have demonstrated the predictive values of distressing symptoms for acute impaired aspects of functioning and eventually increased risk of developing clinical mental disorders [77, 78].

### Clinical implications

A recent global survey on the digitalization of mental healthcare practices reported a low level of usage of mobile applications in screening and diagnosis of mental illness despite the widespread availability [79]. ESM then shows potential for developing early detection systems. Early detection supported by ESM designs shows potential in identifying critical transitions, or “tipping points”, of mental health deterioration, fostering early intervention before a shift toward a clinical state is established [80]. Within the context of developing countries, such as Viet Nam and Rwanda, not only can early detection of mental health disorders reduce healthcare burden on already strained systems, but it also allows for prompt treatment to reduce the pervasive stigma of mental health disorders [81, 82]. The m-Path’s ease of use and high tailoring make it an opportunity to position app-based early detection tools as a low-cost, scalable approach to identifying individuals at risk before symptoms worsen [62]. Timely, real-world monitoring through this method can bridge care gaps and foster proactive responses in Vietnam’s and Rwanda’s evolving digital health landscape.

Mobile apps offer a unique platform for monitoring mental health. A study by Bakker and Rickard [83] found that online self-monitoring is beneficial and can complement face-to-face therapy. The ESM method itself involves self-monitoring, providing individuals with deeper insights into their symptoms and prompting them to seek support when needed [84]. Notably, a reduction in grief symptom severity after two weeks of app-based monitoring suggests that online self-monitoring is clinically useful and may complement face-to-face therapy [45]. ESM-based insights into symptoms, context, contingencies, and daily functioning can also inform momentary self-help modules for everyday life interventions [62]. Within the app-based ESM environment, clinicians can extend basic assessments with periodic reminders, automated supportive messages, or therapeutic exercises to aid bereaved individuals in navigating the fluctuating nature of grief in everyday life, thereby creating just-in-time adaptive interventions [85, 86].

### Potential limitations

Despite methodological innovations in assessing daily PGD reactions, our study is without limitations. Similar to previous studies on PGD, non-probability sampling was used to recruit bereaved participants. This might inflate the estimated prevalence of PGD in our sample, as well as reduce the generalizability of our findings, because bereaved individuals who have been more severely affected are more likely to voluntarily participate [9]. Although existing studies suggest that the ESM method can be acceptable and feasible, prolonged exposure to similar questions multiple times a day places an immense burden on the participants, causing message fatigue and consequently non-random missing data [87, 88]. Unfortunately, a systematic review on ecological assessment found that no unifying variables could be identified across the studies that found high correspondence [89]. Additionally, the potential scale-up of app-based digital screening and diagnosis faces multiple challenges given limited policy and regulations on digital tools in mental healthcare around the world [79]. Digital accessibility and literacy may also influence who is able or willing to participate in an app-based ESM study.

## Conclusion

This will be the first study to cross-culturally assess PGD’s real-time dynamics, developmental trajectory, and predictive factors. This knowledge will contribute to the development of early detection tools, timely interventions, and public awareness efforts aimed at bereavement-related distress, especially in non-Western contexts.

## Data Availability

The data supporting the findings of this study are not yet publicly available as participant recruitment and data collection are still in progress. Data will be made available upon reasonable request after the completion of recruitment and finalization of the dataset.
